# Distal Ileal Atresia in a Preterm Infant with Minor Omphalocele: A Case Report

**DOI:** 10.70352/scrj.cr.25-0294

**Published:** 2025-07-15

**Authors:** Yan-Jun Wu, Kai-Hsiang Hsu, Jin-Yao Lai, Jen-Fu Hsu

**Affiliations:** 1 Department of Pediatrics, Taoyuan Armed Forces General Hospital, Taoyuan, Taiwan; 2 Division of Neonatology, Department of Pediatrics, Chang Gung Memorial Hospital Linkou Branch, Taoyuan, Taiwan; 3 School of Medicine, College of Medicine, Chang Gung University, Taoyuan, Taiwan; 4 Graduate Institute of Clinical Medical Science, Chang Gung University, Taoyuan, Taiwan; 5 Department of Pediatric Surgery, Chang Gung Memorial Hospital, Taoyuan, Taiwan

**Keywords:** minor omphalocele, ileal atresia, case report, congenital abdominal wall defect

## Abstract

**INTRODUCTION:**

Omphalocele is a congenital abdominal wall defect that is characterized by herniation of the abdominal viscera through the umbilical ring. Compared with gastroschisis, omphalocele is less frequently associated with ileal atresia. This report describes a preterm newborn with a minor omphalocele complicated by ileal atresia, a complication that may have been previously underestimated.

**CASE PRESENTATION:**

A 5-day-old preterm male infant (gestational age 34 weeks, birth weight 2005 g) presented with delayed meconium passage and persistent bilious gastric aspirates. Antenatal ultrasound revealed an umbilical cyst without any other anomalies. On day 4, a gastrointestinal series examination revealed dilated small bowel loops and a small-caliber colon. Surgical exploration revealed bowel contents entrapped within a 2.2-cm minor omphalocele. The infant was diagnosed with type IIIa distal ileal atresia and colonic atresia, and end-to-end anastomosis was performed. The patient was discharged at a corrected age of 6 weeks on a hypoallergenic semi-elemental formula (50 mL per meal) and partial parenteral nutrition. He was successfully weaned off parenteral nutrition by a corrected age of approximately 10 months. His weight gain was stable, although it remained at approximately the 3rd percentile, and no obvious neurodevelopmental complications were observed.

**CONCLUSIONS:**

This case highlights the importance of recognizing that even minor omphaloceles can be associated with ileal atresia. In neonates with minor omphaloceles, symptoms of feeding intolerance should prompt consideration of this complication.

## INTRODUCTION

Omphalocele is a congenital abdominal wall defect that is characterized by herniation of the abdominal viscera, including the intestines, liver, or other organs, through the umbilical ring. This defect is typically covered by a membranous sac. The prevalence of omphalocele ranges from 2.1 to 3.8 per 10000 live births and is dependent on maternal age, race, and sex.^1,2)^ Compared with gastroschisis, omphalocele is more frequently associated with additional anomalies, including chromosomal abnormalities and structural malformations affecting the cardiovascular, musculoskeletal, gastrointestinal (GI), urogenital, and central nervous systems. Although intestinal atresia is less commonly observed in infants with omphalocele than in those with gastroschisis,^3)^ cases of omphalocele can still present with intestinal obstruction and an increased risk of ileal atresia, particularly in minor omphaloceles (abdominal wall defect <5 cm). This report describes a preterm newborn with a minor omphalocele complicated by ileal atresia, highlighting this rare but significant association.

## CASE PRESENTATION

**Fig. 1:**
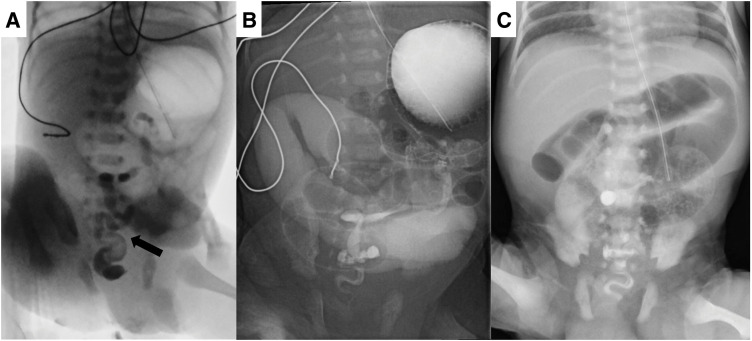
(**A**) A lower GI examination revealed a small-caliber colon (arrow). No luminal dilatation or transitional zones were observed. (**B**) An upper GI examination revealed a patent duodenum with dilated small bowel loops. (**C**) The 1st abdominal X-ray at our hospital revealed dilated bowel loops, suggestive of intestinal obstruction. GI, gastrointestinal

A 5-day-old male preterm (gestational age 34 weeks, birth weight 2005 g) was transferred to our hospital due to delayed meconium passage and bilious gastric aspirates. The mother had an unremarkable antenatal history aside from a risk of pre-eclampsia, for which she began taking aspirin from the 14th week of pregnancy. Antenatal ultrasound had identified an umbilical cyst, although its significance remained unclear at the time. The patient was delivered via emergent Cesarean section due to preterm premature rupture of the membrane and suspicion of placental abruption. His condition was stable after delivery, with Apgar scores of 8 and 9 at 1 and 5 minutes, respectively. Enteral feeding was initiated on day 1 but was soon halted because of the presence of dark bilious aspirates and the absence of meconium passage. On the 4th day after birth, a lower GI series showed a small-caliber colon, while an upper GI series revealed dilated small bowel loops (**Fig. 1A**, and **1B**). The patient was subsequently transferred to our unit for further evaluation and management.

**Fig. 2:**
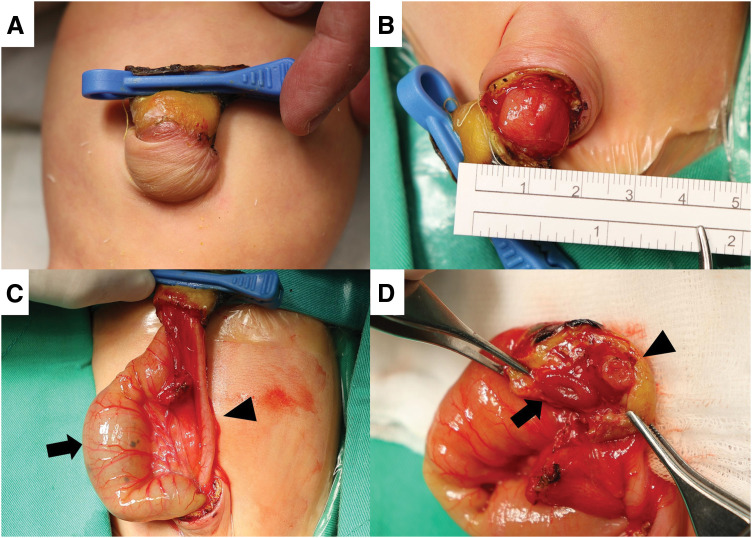
(**A**) Preoperative findings revealed an umbilical mass, which proved to be a minor omphalocele after surgery. (**B**) Bowel contents were found entrapped within the omphalocele (2.2 cm). (**C**) Further exploration of the GI anatomy revealed a significantly dilated proximal ileum (arrow) and collapsed distal bowel loops, likely representing the colon on the basis of the presence of the omentum (arrowhead). (**D**) The section beneath the umbilical clamp revealed a 2-lumen structure, the ileum (arrow) and collapsed colon (arrowhead), which could initially be mistaken for umbilical vessels. GI, gastrointestinal

Upon admission, physical examination revealed marked abdominal distension and an umbilical mass, initially suspected to be an umbilical cord hernia. An X-ray revealed dilated bowel loops and suggested intestinal obstruction (**Fig. 1C**). Although abdominal ultrasonography excluded intestinal malrotation or volvulus, surgical exploration was suggested on the following day (at 6 days of age). The surgeon discovered bowel loops entrapped within a 2.2-cm minor omphalocele, which had been misidentified as an umbilical cord hernia. A proximally dilated ileum and distally collapsed bowel were also noted (**Fig. 2C**), and the ileocecal valve was absent. Pathological report confirmed that the proximal segment was ileal tissue, while the distal collapsed bowel consisted of colonic tissue. A diagnosis of type IIIa ileal atresia with colonic atresia was established. Dissection beneath the umbilical clamp revealed a 2-lumen structure, initially mistaken for umbilical vessels (**Fig. 2D**). Ileal atresia was corrected with an end-to-end anastomosis, and the proximal ileum was tapered by approximately 3 cm to address the luminal size discrepancy.

Enteral feeding was gradually introduced postoperatively. However, the patient subsequently developed abdominal distension accompanied by a significant amount of watery diarrhea. These symptoms persisted despite formula adjustments and pharmacological interventions, necessitating parenteral nutrition. He was discharged at a corrected age of 6 weeks and received a hypoallergenic semi-elemental formula at 50 mL per feeding, along with partial parenteral nutrition and medications for diarrhea control, including loperamide and dioctahedral smectite. By a corrected age of 10 months, the patient was successfully weaned off parenteral nutrition. His weight growth was consistently around the 3rd percentile, and no significant neurodevelopmental impairment was observed.

## DISCUSSION

Omphalocele can be classified into minor or major defects on the basis of the size of the abdominal wall defect, usually with a 5-cm cutoff.^4,5)^ Several studies have highlighted the association between minor omphalocele and intestinal atresia. An earlier study reported that infants with minor omphalocele had a higher incidence of concurrent intestinal anomalies than those with major omphalocele (7/45 [15.6%] vs. 2/59 [3.4%]).^4)^ Another study involving 12 patients with minor omphalocele reported that 4 patients (33%) had GI atresia, including 3 with ileal atresia and 1 with colonic atresia.^5)^ Similarly, in one series involving 53 cases, intestinal anomalies were identified in 6 (11.3%) cases, including Meckel’s diverticulum (4 cases), ileal atresia (1 case), and colonic atresia (1 case). Notably, all of these anomalies were observed in the group with minor omphaloceles.^6)^ These findings underscore a notably higher likelihood of GI anomalies in infants with minor omphaloceles than in those with major omphaloceles.

**Table 1** summarizes the cases of omphalocele coexisting with ileal atresia reported in the literature. Among these reports, Cortese et al.^7)^ described a complex case with multiple intestinal anomalies. While the size of the omphalocele was not mentioned in this report, it was likely to have been a major lesion, as it contained the right lobe of the liver in addition to the ileum. Rekavari et al.^8)^ described a patient who was unable to pass meconium smoothly and was intolerant to enteral feeding. The patient exhibited intestinal obstruction on X-ray and underwent surgery on the 3rd day of life but subsequently died due to sepsis. However, patients with minor or small omphaloceles may also have intestinal atresia, even without specific abdominal symptoms, or exhibit abnormalities on initial X-ray imaging. Salomon et al.^9)^ reported a case of an isolated small omphalocele (2 cm), but surgical exploration unexpectedly revealed the presence of type I intestinal atresia. Another case report described a case of minor omphalocele (2.2 cm) associated with type IV ileal atresia and a congenital ostomy.^10)^ Additionally, Shukri et al.^11)^ and Andargie^12)^ presented cases of either isolated or dual-type ileal atresia coexisting with omphalocele. These reports suggest that even small abdominal wall defects may conceal intestinal atresia. Fortunately, the above-mentioned patients had no other major extraintestinal tract anomalies, and most of the infants survived.

**Table 1:** Summary of our case and previously reported cases of omphalocele coexisting with ileal atresia

Case	Sex	BW (g)	GA (weeks)	Symptom	X ray	Omphalocele size (cm)	Type of atresia	Additional intestinal anomaly	Other major anomaly	Outcomes
Cortese et al.^7)^	M	1700	38	Not mentioned	Dilated stomach, no gas in intestine	Not mentioned	Multiple atresia (pyloric, jejunum, ileum, colon)	Meckel’s diverticulum	Not mentioned	Expired due to heart failure
Rekavari et al.^8)^	F	2600	Not mentioned	Delayed meconium passage, feeding intolerance	Intestinal obstruction	Not mentioned	Type I	No	No	Expired due to sepsis
Salomon et al.^9)^	M	2205	36.5	Not mentioned	Not mentioned	2.0	Type I	Meckel’s diverticulum	No	Survived
Etensel et al.^10)^	F	2120	35	Not mentioned	Not mentioned	2.2	Type IV	Congenital ostomy	No	Survived
Shukri et al.^11)^	F	3200	38	Not mentioned	Not mentioned	3.0	Type I and IIIa	No	No	Survived
Andargie^12)^	F	1800	Not mentioned	Non-specific	Not mentioned	3.5	Type I	No	No	Survived
Our case	M	2005	34	Delayed meconium passage, feeding intolerance	Intestinal obstruction	2.2	Type IIIa	Colonic atresia	No	Survived

BW, birth weight; F, female; GA, gestational age; M, male

The pathogenesis of the association between omphalocele minor and intestinal atresia remains inconclusive. As early as 1962, Grob^13)^ described intestinal strangulation at the umbilical ring, with or without secondary atresia, as one of the complications of omphalocele requiring urgent intervention. Although not directed linked to omphalocele, Pratap et al.^14)^ reported a case of ileal atresia entrapped within the umbilical ring. One review hypothesized that vascular occlusion resulting from mechanical compression at the neck of the omphalocele minor defect could be the cause.^5)^

Furthermore, a study indicated that, compared with omphalocele major, a minor lesion is more prone to be associated with chromosomal or syndromic anomalies, as well as GI and central nervous system abnormalities.^15)^ In a case of omphalocele minor coexisting with complete absence of the large bowel,^16)^ the author proposed that the 2 lesions developed independently, given the diverse blood supply to the large bowel and rectum. Therefore, while mechanical compression leading to vascular occlusion is a potential cause, chromosomal anomalies or syndromic abnormality should also be considered in cases of omphalocele minor associated with intestinal defects.

## CONCLUSIONS

This case highlights the importance of recognizing that even minor omphaloceles can be associated with ileal atresia. In neonates with minor omphaloceles, symptoms of feeding intolerance should prompt consideration of this complication.

## DECLARATIONS

### Funding

Not applicable.

### Authors’ contributions

Yan-Jun Wu wrote the manuscript.

Kai-Hsiang Hsu, Jin-Yao Lai, and Jen-Fu Hsu reviewed the manuscript.

All authors read and approved the final manuscript.

### Availability of data and materials

Not applicable.

### Ethics approval and consent to participate

The Chang Gung Medical Foundation Institutional Review Board approved this case report (No. 202500422B0).

### Consent for publication

Written informed consent was obtained from the parents for publication of this case report.

### Competing interests

The authors declare that they have no known competing financial interests or personal relationships that could have appeared to influence the work reported in this paper.
